# Supplementary material for: The power of one clean qubit in supervised machine learning
Source: Sci Rep. 2023 Nov 15;13:19975. doi: 10.1038/s41598-023-46497-y (PMC10651850; doi:10.1038/s41598-023-46497-y)
Supplement: Supplementary file 1 — Supplementary Information. [file 41598_2023_46497_MOESM1_ESM.pdf]

The Power of One Clean Qubit in Supervised Machine Learning

Mahsa Karimi,[1,2,*] Ali Javadi-Abhari,[3] Christoph Simon,[1,2] and Roohollah Ghobadi[1,2,†]

[1] *Department of Physics and Astronomy, University of Calgary, Calgary, AB, T2N 1N4, Canada*
[2] *Institute for Quantum Science and Technology, University of Calgary, Calgary, AB, T2N 1N4, Canada*
[3] *IBM Quantum, IBM T. J. Watson Research Center*

(Dated: October 25, 2023)

I. DERIVING EQ.7

The input state of the control qubit can be obtained by tracing out Eq.1 in the main manuscript.

$$\rho_{in,c} = \frac{1}{2}\begin{pmatrix} 1 & \alpha \\ \alpha & 1 \end{pmatrix} = \frac{1+\alpha}{2}\left|+\right\rangle\left\langle+\right| + \frac{1-\alpha}{2}\left|-\right\rangle\left\langle-\right|, \qquad (S1)$$

where $|+\rangle = \frac{|0\rangle+|1\rangle}{\sqrt{2}}$ and $|-\rangle = \frac{|0\rangle-|1\rangle}{\sqrt{2}}$. Using the definition of von Neumann entropy $[S(\rho) = -\mathrm{tr}(\rho\log\rho)]$ for Eq.(S1) it is straightforward to obtain $S(\rho_{diag,in,c}) = 1$ and $S(\rho_{in,c}) = H_2\left(\frac{1-\alpha}{2}\right)$, where we used the binary entropy $H_2(x) = -x\log_2 x - (1-x)\log_2(1-x)$. Therefore upon using Eq.6, one obtains

$$C(\rho_{in,c}) = 1 - H_2\left(\frac{1-\alpha}{2}\right). \qquad (S2)$$

Next, we obtain the coherence in the final state Eq.3. Noting that the eigenvalues of Eq.3 are $\mu_\pm = \frac{1\pm|\alpha\,\mathrm{tr}\,U|/2^n}{2}$, we get $S(\rho_{f,c}) = H_2\left(\frac{1-|\alpha\,\mathrm{tr}\,U|/2^n}{2}\right)$. Also, since all the diagonal entries of $\rho_{f,c}$ are equal, we have $S(\rho_{diag,f,c}) = 1$. Thus, we obtain

$$C(\rho_{f,c}) = 1 - H_2\left(\frac{1-|\alpha\,\mathrm{tr}\,U|/2^n}{2}\right). \qquad (S3)$$

Finally, $\Delta C = C(\rho_{in,c}) - C(\rho_{f,c})$ together with Eqs. (S2,S3) conclude

$$\Delta C = H_2\left(\frac{1-|\alpha\,\mathrm{tr}\,U|/2^n}{2}\right) - H_2\left(\frac{1-\alpha}{2}\right), \qquad (S4)$$

which is Eq.7 in the main manuscript.

* mahsa.karimi1@ucalgary.ca
† farid.ghobadi80@gmail.com
